# Catalase Gene Family in Durum Wheat: Genome-Wide Analysis and Expression Profiling in Response to Multiple Abiotic Stress Conditions

**DOI:** 10.3390/plants12142720

**Published:** 2023-07-21

**Authors:** Mouna Ghorbel, Ikram Zribi, Malek Besbes, Nouha Bouali, Faiçal Brini

**Affiliations:** 1 Department of Biology, College of Sciences, University of Hail, P.O. Box 2440, Ha’il City 81451, Saudi Arabia; m.ghorbel@uoh.edu.sa (M.G.); ikram.zribi@cbs.rnrt.tn (I.Z.); m.besbes@uoh.edu.sa (M.B.); n.bouali@uoh.edu.sa (N.B.); 2 Laboratory of Biotechnology and Plant Improvement, Centre of Biotechnology of Sfax, P.O. Box 1177, Sfax 3018, Tunisia

**Keywords:** abiotic stress, durum wheat, gene expression, catalase proteins, in silico analysis, reactive oxygen species

## Abstract

Catalase (CAT) is an antioxidant enzyme expressed by the *CAT* gene family and exists in almost all aerobic organisms. In fact, the CAT enzyme modulates the hydrogen peroxide (H_2_O_2_) contents in cells by translating this toxic compound into water (H_2_O) and O_2_^−^ to reduce reactive oxygen species (ROS) contents in cells. ROS are produced as a result of biotic and abiotic environmental stressors. To avoid ROS toxicity, plants are armed with different enzymatic and non-enzymatic systems to decompose ROS. Among the enzymatic system, CAT proteins are well studied. CAT not only controls growth and development in plants but is also involved in plant defense against different stresses. So far, the *CAT* gene family has not been reported in durum wheat (*Triticum turgidum* ssp. *durum* L.). Therefore, a genome-wide comprehensive analysis was conducted to classify the *CAT* genes in the durum wheat genome. Here, six *TdCAT* genes were identified. Based on phylogenetics, the *TdCAT* genes belong to three groups (Groups I–III) which is explainable by their comparable structural characteristics. Using bio-informatic analysis, we found that the secondary and tertiary structures were conserved among plants and present similar structures among durum wheat CATs. Two conserved domains (pfam00199 and pfam06628) are also present in all identified proteins, which have different subcellular localizations: peroxisome and mitochondrion. By analyzing their promoters, different cis-elements were identified, such as hormone-correlated response and stress-related responsive elements. Finally, we studied the expression pattern of two catalase genes belonging to two different sub-classes under different abiotic stresses. Expression profiling revealed that TdCAT2 and TdCAT3 presented a constitutive expression pattern. Moreover, both genes are induced in response to salt, mannitol, cold, heat and ABA. Thus, we speculate that those genes are activated by different stresses, such as oxygen deficiency, light, cold, abscisic acid and methyl jasmonate. Further, this study will help in understanding the behavior of *CAT* genes during environmental stress in durum wheat and in *Triticeae* species in general.

## 1. Introduction

Abiotic (such as flooding, drought, cold, and high temperature) and biotic (bacteria, viruses, fungi, nematodes…) stressors have an impact on plant growth and development [1]. Those stresses are important sources of Reactive oxygen species (ROS) in cells [1]. In many biological processes, ROS serve as signaling molecules, but an excessive buildup of ROS in living plants can cause oxidative stress damage to cells [2,3]. In fact, environmental constraints modify cellular redox homeostasis, inducing the over-production of small toxic compounds (ROSs), such as hydroxyl radicals (OH^−^), superoxide (O_2_^−^), hydrogen peroxide (H_2_O_2_), and other oxygen radicals which alter the cellular redox equilibrium [4,5,6]. These extremely hazardous ROSs needs to be changed into less reactive forms in order to adapt to the living environment. To defend themselves from oxidative damage, plants have developed efficient detoxification mechanisms, including non-enzymatic and enzymatic detoxification systems [4]. ROSs act as important second messengers in many signaling pathways, such as the increase of endogen abscisic acid (ABA) levels [7] and different other stresses [8,9]. The diverse types of antioxidant enzymes found in enzymatic systems serve as the primary mechanisms for eliminating ROSs. Due to their high affinity for H_2_O_2_, catalases (CATs) are regarded as the most effective ROS scavengers [5,6]. Nearly all living organisms possess CATs, which have been extensively studied [10]. Numerous studies have demonstrated that the regulation of plant *CAT* gene expression affects how the organism develops, matures, and responds to environmental cues [11,12,13]. In *Arabidopsis thaliana*, *AtCAT* genes encode for a small family of proteins (three isoforms known as AtCAT1, AtCAT2, and AtCAT3), which catalyze the oxidation of H_2_O_2_ and are crucial for maintaining the equilibrium of ROSs [14]. The *AtCAT1* gene expression is increased by ABA through the MAPK cascades [15,16], but it does not appear to be affected by circadian rhythms. *AtCAT2* is mostly expressed in leaves and can be stimulated by light, cold, and possibly even the circadian rhythm [16]. Under typical growth conditions, the *AtCAT2* mutant (*atcat2*) and only 20% of the wild-type leaf catalase activity accumulate more H_2_O_2_ than the wild-type [17]. *AtCAT3* gene, engaged in ABA-mediated stomatal regulation in response to drought stress [7], is controlled by CPK8 and exhibits high levels of expression throughout the entire plant at all developmental stages [18]. Different other CAT proteins are also isolated from other plants. Three *CAT* genes, *OsCATA*, *OsCATB*, and *OsCATC*, have been discovered in rice [9]. The most stress-responsive members of *OsCAT genes* are *OsCATA* and *OsCATC*, according to earlier research [9]. In durum wheat (*Triticum turgidum* ssp. *durum*), the first isolated *CAT* gene (*TdCAT1*) from the wheat genome (cv. Om Rabiaa, a local Tunisian variety) is highly expressed in the whole plant at all developmental stages [13]. The same findings were observed in the Waha ecotype, a Saudi variety [14], and in *Triticum monococcum* [15]. Moreover, TdCAT1 is regulated by bivalent cations and by the calcium/Calmodulin complex [16]. Further, TdCAT1 harbors an autoinhibitory domain located at the C-terminal portion of the protein [17]. Interestingly, no other catalase gene was identified in the durum wheat genome.

Durum wheat (*Triticum durum* Desf.) is an important source of food around the globe, with an estimated 36 million tons of annual global production [18]. Turkey and Canada are the top two producers, with an estimated 2 million ha each [18]. India, Algeria, and Italy follow, each cultivating more than 1.5 million ha [19,20,21]. Durum wheat is grown on 0.5 to 0.8 million ha annually in France, Greece, Morocco, Pakistan, Portugal, Kazakhstan, Russia, Spain, and Tunisia [18], with approximately 0.6 million ha, Ethiopia is the largest producer of durum wheat in Sub-Saharan Africa (SSA) [22]. This crop has a very diverse genetic heritage, and this diversity is reflected in the numerous traditional ways that it is consumed, including a number of distinctive dishes that proudly embody the national identities: pasta, couscous, bourghul, freekeh, gofio, and unleavened bread, to name a few local dishes [22]. Durum wheat is currently grown in affluent countries primarily as a commercial crop to fuel the burgeoning food Industry, despite its close ties to traditional recipes [22]. The most profitable crops are durum wheat and rice, with prices typically 20 to 40% higher than those of common wheat, millet, maize, and sorghum [22]. Due to its outstanding resistance to climatic pressures, durum wheat continues to be an essential staple crop for smallholder farmers on marginal lands, but its large-scale production is closely correlated with its higher financial return.

In contrast to the research progress in other species, knowledge of the *CAT* genes in wheat is still limited. Therefore, in this study, a comprehensive genome-wide analysis of *CAT* genes in durum wheat was carried out. Indeed, phylogenetic relationships, the in silico subcellular localization, conserved domains, gene structures, gene locations, and cis-elements in the promoters of *TdCAT* genes were fulfilled. In addition to these latter, relative expression levels of two *TdCAT* genes had shown remarkable changes in response to different stress treatments, as well as plant treatment with ABA phytohormone.

## 2. Results

### 2.1. Identification, Alignment, Gene Structures, Distribution, and Conserved Motifs of CAT genes in Triticum durum

**Figure 1:**
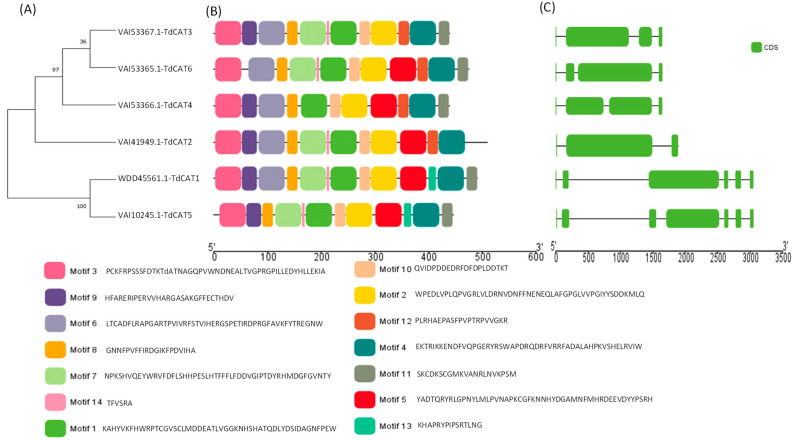
Identification, Gene Structures, Distribution, and Conserved Motifs of *CAT* genes in *Triticum durum* genome. (**A**) Construction of phylogenetic tree using Mega 11 showing that TdCAT proteins are clustered in three main clusters; (**B**) Analysis of conserved motifs in TdCAT proteins using MEME server and visualization with TbTools v1.123; (**C**) Genes structures presentations using TbTools. The abscissa in (**B**,**C**) represents the length of proteins and genes, respectively. The green rectangle presents the CDS of genes in (**C**).

After Running of Blast program (Blast+2.14.0), 25 sequences resulted from the alignments of the durum wheat genome and the conserved domain PF00199. The outcoming of several programs asserted their possession of the functional CAT domain (PF00199.12) and catalase-related domain (PF06628.5). Grounded on the Decrease redundancy program (https://web.expasy.org/decrease_redundancy/ (accessed on 10 April 2023)), six *CAT* genes were identified in durum wheat (named *TdCAT1* to *TdCAT6*; Figure 1).

In order to understand the evolutionary relationship between CAT proteins, a phylogenetic tree was constructed using the six TdCAT proteins identified in the durum wheat genome (Figure 1A, Supplementary Figure S1). The phylogenetic tree showed that TdCAT proteins are subdivided into three different clusters. TdCAT1 and TdCAT5 are clustered in the same group. TdCAT2 forms another group, whereas the third group is formed by TdCAT3, TdCAT4 and TdCAT6.

The Multiple Em for Motif Elicitation (MEME) database (version 5.5.1) was used to identify the putative motifs in the TdCAT protein sequences. Interestingly, fourteen motifs were identified. Six of them were presented in all the selected TdCAT proteins (Motif 1, 2, 3, 4, 8 and 10), whereas motif 13 was identified only in TdCAT1 and TdCAT5 belonging to the same cluster. Motif 7 (presented by light green boxes), motif 9 (presented by purple boxes) and Motif 11 (presented by grey boxes) are present in all TdCAT proteins except in TdCAT3, TdCAT2 and TdCAT4, respectively (Figure 1B). Furthermore, the analyses of the exon–intron organization of *TdCAT* genes were performed to understand the evolution of these genes. Analyses depicted that durum wheat *CAT* genes presented different structures. In fact, *TdCAT1* and *TdCAT5*, belonging to the same cluster, present 6 exons and 5 introns, respectively, whereas the other genes (*TdCAT3, TdCAT4* and *TdCAT6*) belonging to the second cluster harbor 4 exons and 3 introns, respectively. Besides, only *TdCAT2* presents 3 exons and 2 introns (Figure 1C). The genomic features of *TdCAT* genes are presented in Table 1.

**Figure 2:**
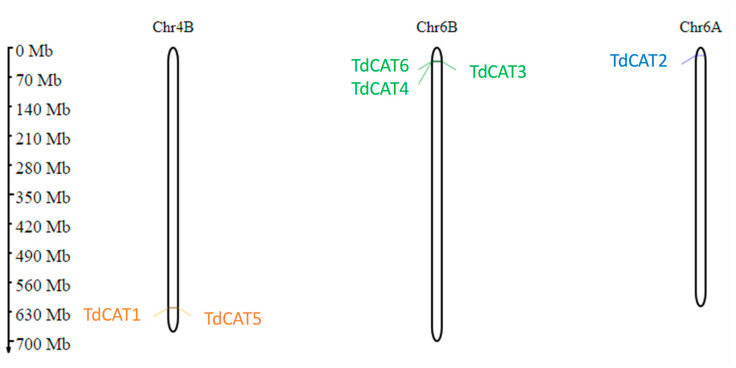
Prediction of *TdCAT* genes chromosomal localization in *Triticum durum* genome using MG2C. Classification was based on their groups I, II, and III. Gene ID are colored in orange, green, and blue, respectively.

The distribution of these genes was observed on three different chromosomes. In fact, two genes (*TdCAT1* and *TdCAT5*) are located on Chr4B, one gene (*TdCAT2*) is located on chromosome 6A and the other three genes (*TdCAT3*, *TdCAT4* and *TdCAT6*) are located on Chr6B (Figure 2, Table 1).

**Figure 3:**
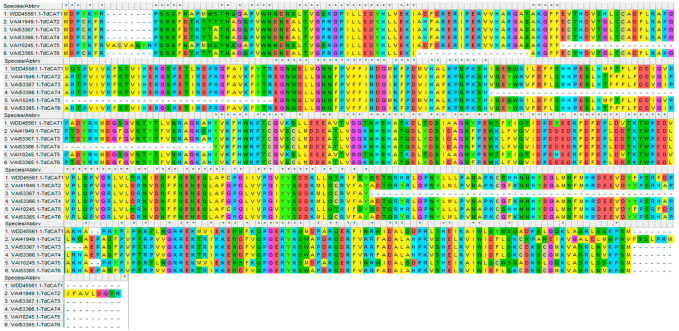
Sequence alignment of the six TdCAT proteins using MEHGA11 software (Muscle). The two conserved domains pfam00199 and pfam06628 were represented by red and blue line, respectively.

Moreover, the multiple alignments performed using the Muscle algorithm showed that TdCAT protein sequences are homologous (Figure 3).

Further bioinformatic analysis showed that durum wheat catalase proteins do not present cleavage sites in their structures as revealed by Protter server (Supplementary Figure S2) which are similar to the outcomes found previously on the TdCAT1 protein, the first isolated catalase from durum wheat [14]. A glycosylation site was identified for all identified proteins. These sites are located at different parts of the proteins: Asn-247 (for TdCAT1, TdCAT2 and TdCAT3), Asn-198 (for TdCAT4), Asn-201 (for TdCAT5) and Asn-228 (for TdCAT6) (Table 2).

**Figure 4:**
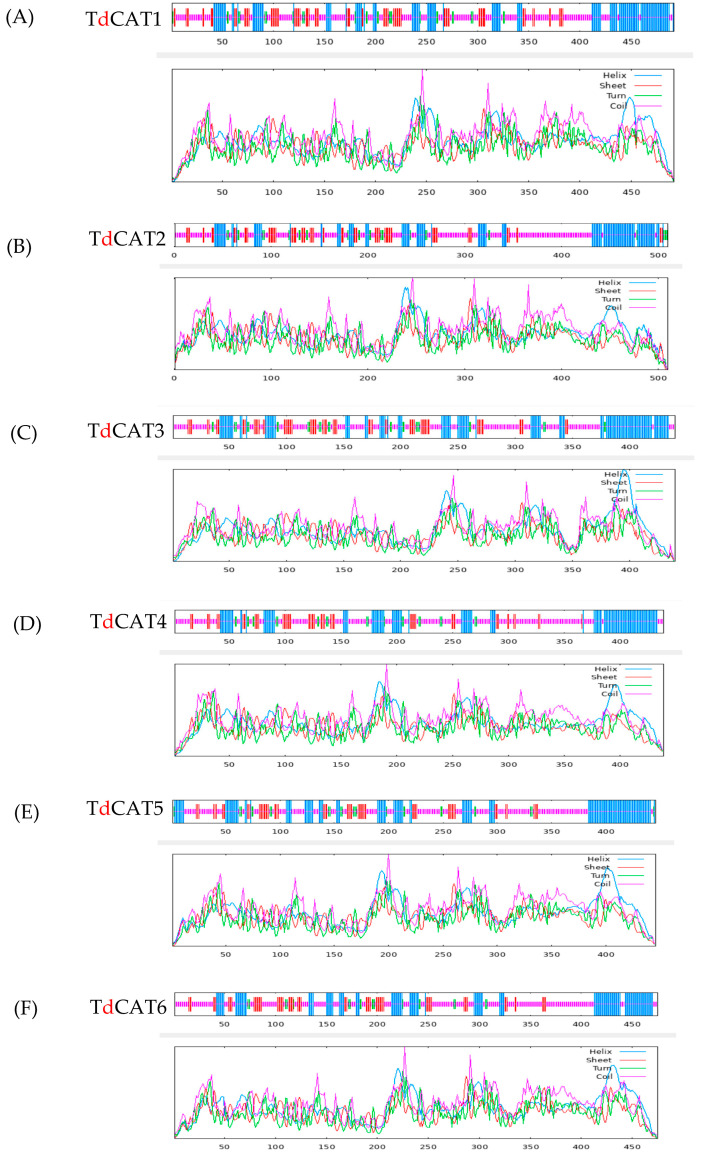
2D structures of the six identified TdCATs in durum wheat genome (**A**) TdCAT1 structure; (**B**) TdCAT2; (**C**) TdCAT3; (**D**) TdCAT4; (**E**) TdCAT5 and (**F**) TdCAT6.

Finally, TdCAT proteins were used to search the number of phosphorylated sites in the proteins using NetPhos software. As shown in Figure 4A, the number of phosphorylated sites in TdCAT proteins varies from 31 in TdCAT3 and TdCAT4 to 40 in TdCAT1 suggesting the importance of phosphorylation in TdCAT activities. Moreover, all identified proteins presented disordered regions: 14.5% in TdCAT3 to 16.25% to TdCAT5 (Supplementary Figure S3). In addition, the sequences of the identified catalase proteins were analyzed to look for the presence of transmembrane helices. Interestingly, no transmembrane region was found in all identified catalase proteins (Supplementary Figure S2).

### 2.2. Identification of the 2D and 3D Structures of Catalases in Triticum durum

The secondary (2D) structure of TdCAT proteins was predicted using the SOPMA programs. All identified proteins revealed alpha helix, beta turns, extended strand, and random coil. These structures were represented by small lines of different colors (Table 3; Figure 4).

Interestingly, the arrangement of these secondary structures in all proteins was very similar. For all identified catalase proteins, random coil accounted for a large proportion (47–54%). The second was alpha-helix (26–30%) and those components were concentrated on the N-terminal region of the proteins. Beta turns formed the smallest proportion (4–6%) of secondary structures whereas the extended strands counted between 13 and 16%.

**Figure 5:**
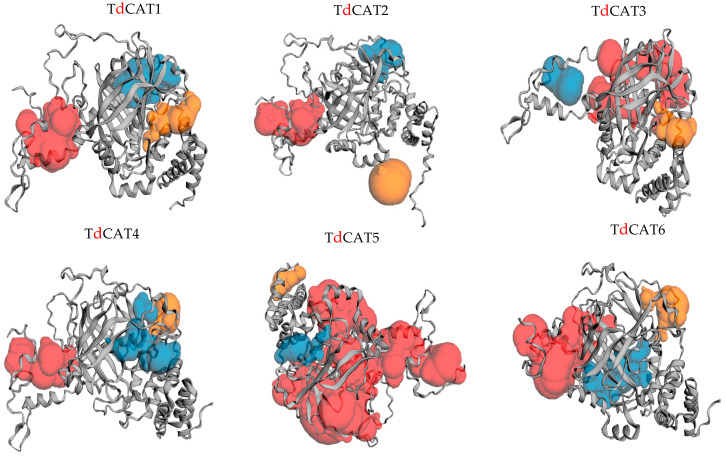
Prediction of pockets presented in the 3D structure of the TdCAT proteins using the CASTp server. The top three predicted pockets are indicated as red, blue and orange, respectively.

Alphaflod was used to modulate the 3D structures of the proteins (Figure 5). The TdCAT tridimensional proteins structures have some differences between each other’s. The structure of TdCAT5 slightly differs from other proteins. Such diversities in the protein structures may reflect their different functions. According to the CASTp 3.0 analysis, molecular pockets were identified in all proteins. The top three predicted pockets, with the largest volume, are indicated as red, blue, and yellow, respectively (Figure 5). 

### 2.3. Phylogenetic Analysis of TdCATs

**Figure 6:**
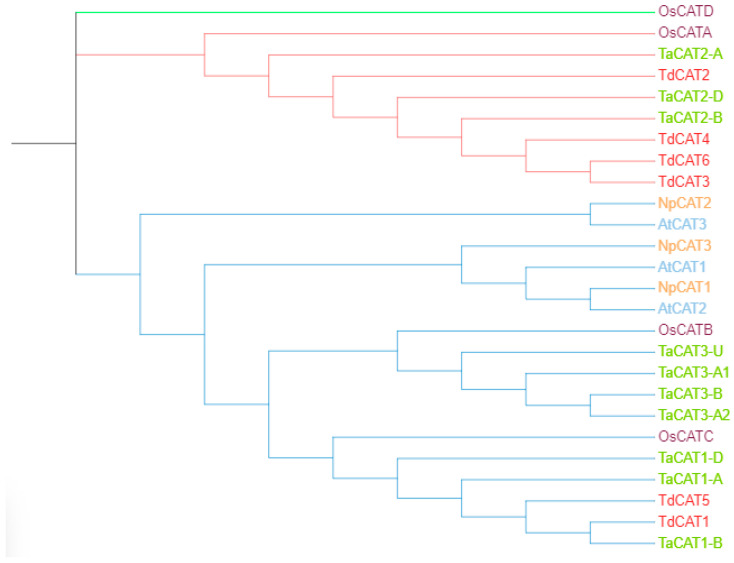
The phylogenetic tree of CAT proteins from different species *Triticum turgidum* ssp. *durum*, *Oryza sativa* ssp. *Japonica* (OsCATA: XP_015625395; OsCATB: XP_015643077; OsCATC: Q10S82.1; OsCATD XP_015636098.1), *Nicotiana plumbaginifolia* (NpCAT1: P49315.1; NpCAT2: P49316.1; NpCAT3: P49317.1), *Arabidopsis thaliana* (AtCAT1: AAQ56816.1; AtCAT2: AAL66998.1; AtCAT3: NP_564120.1) and *Triticum aestivum* (TaCAT1-B: TraesCS4B02G325800; TaCAT1-D: TraesCS4D02G322700; TaCAT1-A: TraesCS5A02G498000; TaCAT2-A: TraesCS6A02G04170; TaCAT2-B: TraesCS6B02G056800; TaCAT2-D: TraesCS6D02G048300; TaCAT3-A1: TraesCS7A02G549800; TaCAT3-A2: TraesCS7A02G549900; TaCAT3-B: TraesCS7B02G473400; TaCAT3-U: TraesCSU02G105300) was constructed with test maximum likehood with 1000 bootstraps by MEGA 11.

An unrooted phylogenetic tree was constructed using MEGA 11 software with CAT proteins from *T. aestivum*, *Oryza sativa* ssp Japonica, *Nicotiana plumbaginifolia* and *Arabidopsis thaliana*; Figure 6). According to the phylogenetic tree, twenty-six *CAT* genes were divided into three different classes (class I, class II, and class III). The first group (represented by blue color) is formed by TdCAT1 and TdCAT5 together with TaCAT1-A, TaCAT1-B, TaCAT1-D, TaCAT1-A, TaCAT1-D, TaCAT3-A2, TaCAT3-B, TaCAT3-A1, TaCAT3-U and OsCATC together with other catalase from Arabidopsis and tobacco. The second group (Represented in red) is formed exclusively by monocotyledonous proteins (*Oryza sativa, Triticum aestivum* and *Triticum turgidum* ssp. *durum*) (TdCAT2, TdCAT3, TdCAT4 and TdCAT6). Finally, the third group was formed by the rice catalase OsCAT-D.

### 2.4. Identification of CaM Binding Domains

We have recently characterized a CaM binding domain (CaMBD) located at the C-terminal portion of the TdCAT1 protein [16]. This domain was also conserved in different other proteins and ensures the interaction of the CaM/Ca^2+^ complex with the TdCAT1 protein in Calcium independent manner and stimulates the catalytic activity of the protein in Calcium dependant manner. To investigate whether other identified TdCAT1s harbor such a domain, we analyzed the structure of the 6 TdCAT1 proteins using the calmodulin target database. As revealed in Table 3, all identified TdCAT proteins harbor at least 3 putative CaMBDs located at different parts of the proteins. Interestingly, all identified catalase proteins harbor an IQ motif (Table 4, Supplementary Figure S4). The biological significance of such domains remains unclear.

### 2.5. In Silico Localization of TdCAT Proteins

The subcellular localization of TdCAT proteins was also performed using different servers. The results showed that TdCATs presented different subcellular localizations as shown in Table 5. In fact, CELLO2GO and Pannzer2 online tools display that these proteins are peroxisomal, whereas Wolf PSORT illustrated that they could be peroxisomal, cytoplasmic or chloroplastic proteins.

### 2.6. Gene Ontology (GO) Term Distribution of Triticum turgidum ssp. durum Catalase

To identify the biological process and the molecular functions of the different isolated proteins, Pannzer2 tool was used. The results, represented in Table 6, showed that all identified proteins regulate the hydrogen peroxide catabolic process and cellular oxidant detoxification. All proteins are also implicated in plant response to abiotic stimulus and to hormones. TdCAT2, TdCAT3 and TdCAT6 control the circadian rhythm of the plants and to their response to salt stress. TdCAT1, TdCAT3, TdCAT4 and TdCAT5 are implicated in plant response to oxidative stress. TdCAT1 and TdCAT5 are implicated in plant response to cadmium. Finally, TdCAT2 and TdCAT6 are responsive to ROS whereas TdCAT1 and TdCAT5 are implicated in protein nitrosylation.

### 2.7. In Silico Analysis of Cis-Elements

**Figure 7:**
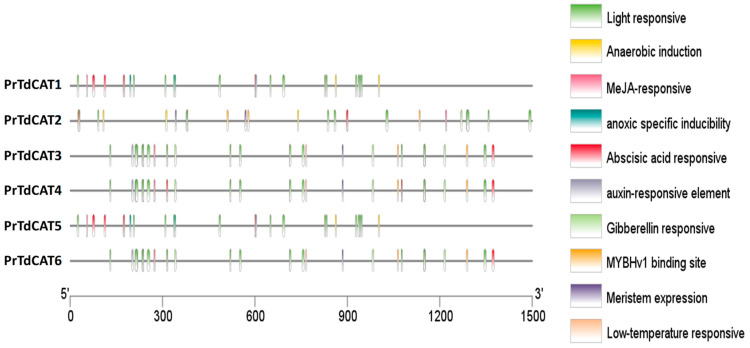
Analysis of cis-acting elements of the putative *TdCAT* promoters using the PlantCARE online server and visualized by Tbtools.

To further understand cis-elements of different genes, the 1.5 kb 5′ upstream region of the 6 *TdCAT* genes was investigated using the PlantCARE database. In addition to some basic core components, the results showed the presence of different cis-acting element identified in the *TdCAT* promoters could be divided into three categories, such as development-related elements (meristem expression) environmental stress-related elements (such as light responsive and low temperature responsive), and anoxia or anaerobic induction elements, and hormone responsive elements (such as MeJA responsive, abscissic acid responsive (ABRE), auxin responsive and Gibberelline responsive elements) (Figure 7; Supplementary Figure S5). In addition, a serie of elements, such as G-box (Sp1), MeJA-response element, and abscissic acid responsive (ABRE) are known for their important roles as key components of abiotic stress responsiveness. Those elements were common to all *TdCAT* genes suggesting that those genes could play a crucial role in different abiotic stresses. For exemple, anaerobic induction elements are identified in the promoter of *TdCAT1* and *TdCAT5*, wherease LTR (Low temperature responsive) are detected in the promoters of *TdCAT3*, *TdCAT4* and *TdCAT6*. Moreover, all promoters contain at least one element related to meristem expression, except for *TdCAT1* and *TdCAT5*, indicating that these genes may be related to meristem development. In addition, all promoters contain also one element for auxin responsive elements (TGA-elements) except for *TdCAT2*. In the light of those findings, we can speculate that *TdCAT* genes of the same class may have different modes of action, and that genes of different classes may work together.

**Figure 8:**
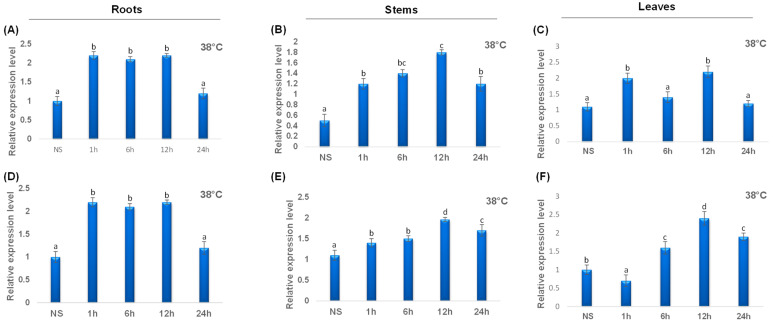
QRT-PCR expression analysis of *TdCAT2* and *TdCAT3* genes under heat treatment (38 °C). Relative expression levels of TdCATs in response to heat treatment for non-stressed (0 h), 1 h, 6 h, 12 h, and 24 h at the 10 days old in the roots (**A**,**D**), stems (**B**,**E**) and leaves (**C**,**F**) of *TdCAT2* and *TdCAT3* genes, respectively. Data were normalized with *actin* gene, and vertical bars indicate standard deviation error. Different letters indicate significant differences at *p* < 0.05 according to one-way ANOVA and post-hoc Tukey’s test. NS means non stressed plants.

To point out the transcript levels of the *TdCAT* genes, we examined three organs of durum wheat (roots, stems and shoots) at 10 days growth stage and different stress conditions. When the gene expression patterns were analyzed, it became clear that the *TdCAT* genes belonging to the same subgroups were similarly expressed. *TdCAT2* presented the lowest transcript level in stems at normal plant culture conditions as previously demonstrated for *TaCAT2B* (Figure 8). *TdCAT2* was induced under heat treatment (38 °C) in all investigated tissues after 1 h of stress application. In roots, this induction remains approximatively constant and decreased after 24 h of stress application to reach the normal values (Figure 8A), whereas, in stems, the expression level of *TdCAT2* gene increased gradually and reached its maximum 12 h after stress application then dropped (Figure 8B). In leaves, the expression level increased after 1 h of stress application then decreased at 6 h after stress. Interestingly, this expression increased 12 h after stress application to decrease again after 24 h (Figure 8C). Moreover, *TdCAT3* gene was also induced in response to heat stress (Figure 8). *TdCAT3* was detected in all tissues at that stage (10 days old) and presented a constitutive expression pattern. In addition, in response to heat stress, *TdCAT3* presented the same expression pattern as *TdCAT2* in roots and stems (Figure 8D,E). In leaves, the expression of *TdCAT3* was slightly repressed after 1 h of stress application then increased gradually to reach its maximum after 12 h of stress application and then decreased after 24 h of stress application (Figure 8F).

**Figure 9:**
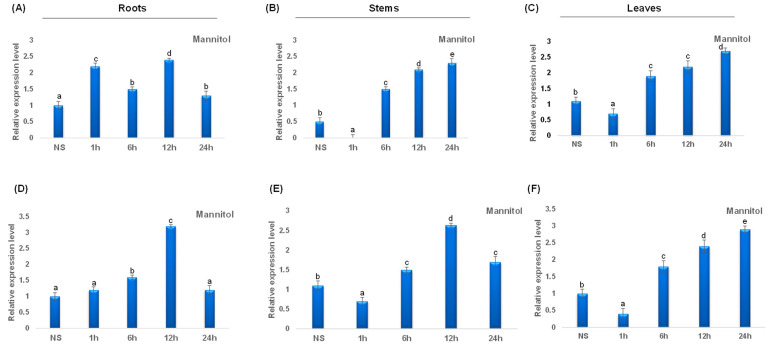
QRT-PCR expression analysis of *TdCAT2* and *TdCAT3* genes under mannitol treatment (150 mM). Relative expression levels of *TdCATs* in response to Mannitol treatment for non-stressed (0 h), 1 h, 6 h, 12 h, and 24 h at the 10 days old in the roots (**A**,**D**), stems (**B**,**E**) and leaves (**C**,**F**) of *TdCAT2* and *TdCAT3* genes, respectively. Data were normalized with *actin* gene, and vertical bars indicate standard deviation error. Different letters indicate significant differences at *p* < 0.05 according to one-way ANOVA and post-hoc Tukey’s test. NS means non stressed plants.

Under mannitol stress, *TdCAT2* was repressed in leaves after 1 h of stress application, then the expression level increased considerably after 6 h of stress and remains elevated after 24 h of stress application. The same result was also observed in stems and roots (Figure 9A–C). Besides, *TdCAT3* gene was also induced after mannitol stress application in roots. This induction was observed for the first 12 h then a decrease in *TdCAT3* transcript was observed. In stems, *TdCAT3* gene was repressed after 1 h of stress and reaches its maximum after 12 h of stress application then decreased. In leaves, the same effect was also observed but the expression level remains elevated after 24 h of stress application. 

**Figure 10:**
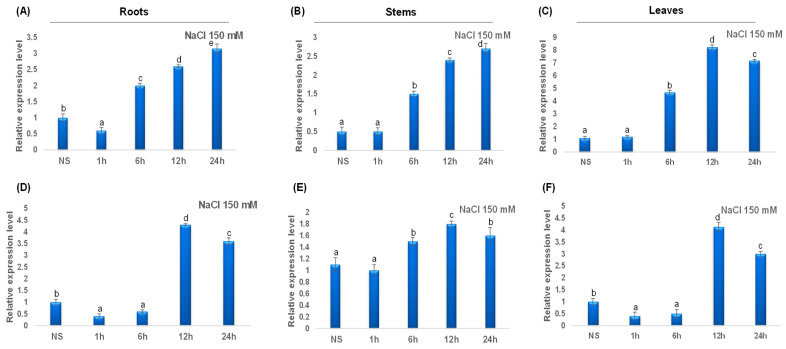
QRT-PCR expression analysis of *TdCAT2* and *TdCAT3* genes under NaCl treatment (150 mM). Relative expression levels of *TdCATs* in response to NaCl treatment for non-stressed (0 h), 1 h, 6 h, 12 h, and 24 h at the 10 days old in the roots (**A**,**D**), stems (**B**,**E**) and leaves (**C**,**F**) of *TdCAT2* and *TdCAT3* genes, respectively. Data were normalized with *actin* gene, and vertical bars indicate standard deviation error. Different letters indicate significant differences at *p* < 0.05 according to one-way ANOVA and post-hoc Tukey’s test. NS means non stressed plants.

During salt treatment, *TdCAT2* gene expression decreased in roots (Figure 10A) and remains constant in stems and leaves during (Figure 10B) the first hour of stress treatment then increased in all tested tissues after 6 h of stress to reach their maximums after 12 h in leaves but continues to decrease in roots and stems (Figure 10). For *TdCAT3*, a slight increase in *TdCAT3* gene expression was detected in steam to reach its maximum after 12 h of stress application and then decreased (Figure 10E). In roots and leaves, *TdCAT3* was down-regulated during the first 6 h then up-regulated at 12 h after stress application to drop again at after 24 h (Figure 10D,F).

In addition, *TdCAT2* and *TdCAT3* genes expressions were investigated under low temperature conditions (Supplementary Figure S6). As seen in Figure S6, *TdCAT2* was insensitive to cold stress in all tested tissues whereas *TdCAT3* was down regulated in roots and stems but upregulated in leaves after 6 h of stress then down regulated. 

**Figure 11:**
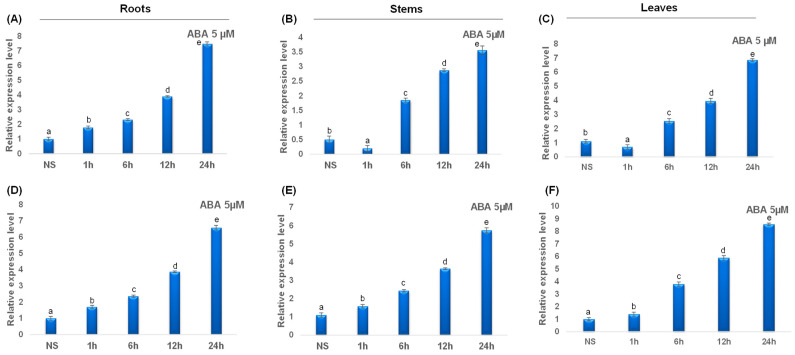
QRT-PCR expression analysis of *TdCAT2* and *TdCAT3* genes under ABA stress treatment (5 μM). Relative expression levels of *TdCATs* in response to ABA treatment for non-stressed (0 h), 1 h, 6 h, 12 h, and 24 h at the 10 days old in the roots (**A**,**D**), stems (**B**,**E**) and leaves (**C**,**F**) of *TdCAT2* and *TdCAT3* genes, respectively. Data were normalized with actin gene, and vertical bars indicate standard deviation error. Different letters indicate significant differences at *p* < 0.05 according to one-way ANOVA and post-hoc Tukey’s test. NS means non stressed plants.

Under ABA treatment, all studied catalase genes were upregulated. In fact, ABA treatment induced a rapid upregulation of *TdCAT2* gene after 1 h of stress application up to 24 h of stress. Interestingly, in steam and leaves, this gene was down-regulated after 1 h of stress application then up-regulated gradually (Figure 11A–C). Interestingly, *TdCAT3* gene was also upregulated in all tested tissues and in all tested times (Figure 11D–F). The data presented here suggest that TdCATs may play crucial roles in durum wheat growth, development, and abiotic stress environment by playing a powerful role in ROS scavenging under different stress conditions.

## 3. Discussion

The catalase gene family is usually small. Genome-wide investigations of CAT families have been widely conducted as the genomes of numerous animals have been sequenced. The number of identified catalase genes in plants varies from one gene in Scots pine [23], three genes in *Arabidopsis* [10], *N. plumbaginifolia* [24], and pumpkin [25], four genes in rice [12], and cucumber [26], seven genes in *N. tabacum* [27], and cotton [28] and ten genes in bread wheat [29].

In order to create novel wheat cultivars with improved resistance to a variety of environmental stresses, it is necessary to understand the biological activities of *TdCAT* genes and the molecular mechanisms underlying their responses to stressful situations. However, due to the intricacy of the wheat gene, the CAT family in wheat has not been adequately studied. In this study, a comprehensive genome-wide analysis of *CAT* genes in durum wheat was carried out. According to their structure/functions of *CAT* genes in plants, CATs are generally divided into three different groups related to vascular, photosynthetic, and reproductive functions [24,25,26,27].

In this work, six *CAT* genes were identified in durum wheat genome and divided into three different classes (Figure 1A). Among the identified genes, three genes were located on the chromosome 6B (*TdCAT3*, *TdCAT4* and *TdCAT6*). The *TdCAT1* and *TdCAT5* genes were mapped into chromosome 4B while *TdCAT2B* was located on chromosome 6A (Figure 2). Interestingly, through sequence alignment, results showed that the similarity of two protein sequences in each class is very high (>96%) (Figure 3) as previously shown for CAT identified from *N. plumbaginifolia* [24]. Such result showed that *CAT* genes are highly conserved during plants evolution (Figure 2 and Figure 3). In bread wheat, the identified genes (10 genes) were located on nine different chromosomes [29]. The same classification was also observed in *N. plumbaginifolia* [24]. In *Nicotiana plumbaginifolia*, NpCat1, NpCat2 and NpCat3 belonged to class I, class II, and class III, respectively. In *Arabidopsis*, AtCAT2 belongs to class I [30]. Similarly, our analyses showed that durum wheat CATs are also classified into 3 different classes (Figure 1A). Moreover, phylogenetic analyses of TdCATs and CATs from other monocotyledonous and dicotyledonous species showed that all those CATs are classified into three different groups with one group harboring monocotyledonous CAT exclusively (Figure 6). *CAT* genes identified in durum wheat, as well as other *CATs* identified in bread wheat, *Arabidopsis* and rice were used to build a phylogenetic tree. The evolutionary tree analysis showed that TdCAT2, TdCAT3, TdCAT4 and TdCAT6 were closely related to catalase identified in bread wheat (Figure 5). Such results suggest that CAT proteins could have specific functions. Moreover, we can suggest that the more closely related genes had a higher similarity in gene structure, but there was some variation among individual genes. In addition to the phylogenetic relationships, conserved domains, gene structures, secondary and tertiary structures, gene locations, in silico proteins localisations, cis-elements in promoters, and relative expression patterns of *TdCATs* were investigated.

Gene structure has been identified as one of the representative traces of gene family evolution [31]. Thus, the detailed structure of *TdCAT* genes was analyzed to study their exon–intron organization (Figure 2). The exons and introns of *TdCAT* genes were found to be different between monocots and dicots as previously shown for bread wheat [29]. In fact, all identified genes presented an important variation among themselves in terms of their intron/exon number and length (Figure 1C).

All *TdCAT* genes possessed two to six exons and three to five introns (Figure 1C). The presence of large introns in TdCAT transcripts may ameliorate the recombination frequency and maintain the counterbalance of mutation bias [12]. In bread wheat, catalase genes presented one to seven introns and two to seven exons [29]. Moreover, a previously identified ancestral copy of a *TaCAT* gene presented seven introns [10]. Different splicing was found in *Arabidopsis* family members. In fact, two kinds of splicing were found in *Arabidopsis* AtCAT2, whereas four kinds of splicing were found in AtCAT3 and a total of seven proteins were encoded by *CAT* genes in *Arabidopsis*. In rice, OsCATA and OsCATB presented three and two kinds of variable shearing. Recently, a novel catalase gene member was found in rice genome named OsCATD. This gene encodes for a long protein of 2392 aa, the longest of all identified catalase genes Thus, this gene formed alone a clade in the phylogenetic tree (Figure 6). Interestingly, protein sequence analysis of OsCATD showed that this protein presented an AMP-binding domain (PF00501.21), a characteristic of OsCATD that was never found in other catalase proteins [12].

The 2D and 3D structures of the proteins were also investigated using SOPMA server. As seen in Figure 4, the structures of all identified TdCAT proteins were predominantly formed by random coils which formed approximately half of the proteins structures followed by the alpha helixes which were concentrated on the C-terminal region of the proteins. Such results were also shown for tobacco catalase proteins [27]. All identified catalase proteins harbor a N-glycosylation site in their structures (Supplementary Figure S2; Table 2). In eukaryotes, N-glycosylation is one of the most crucial protein modifications. This modification controls multiple roles in modulating plant stress tolerance. Recently, mutations in *alg3-3* and *cgl1-1*, involved in N-glycosylation process causes an obvious decrease in photosynthesis in *Arabidopsis* [32]. Moreover, N-glycosylation is required for maintaining CAH1 protein stability. CAH1 is a chloroplast-located protein implicated in photosynthesis suggesting a crucial role of N-glycosylation in regulating photosynthetic efficiency. N-glycosylation is also important for protein folding and transport. In plants, it is also important for the development of stomata [33]. Interestingly, it has been recently shown that mutation in *STT* gene (*stt3a-2* mutant) causes a greater transpiration rate causing an important water loss in plants and an abnormal stomatal distribution. Thus, plants are more susceptible to drought stress [33]. Such phenotype was related to low levels of abscisic acid and auxin in those mutants. All those phenotypes were related to an under glycosylation of AtBG1, a β-glucosidase protein controlling the transformation of conjugated IAA/ABA to active hormones confirming that N-glycosylation process is crucial for stomatal development in plants, as well as in controlling the release of active hormones to regulate plant response to abiotic stresses [33]. In plants, the role of this conserved site in CAT plants is still unknown.

We have recently shown that durum wheat CAT (TdCAT1) harbors a conserved calmodulin binding domain located at the C-terminal portion of the protein [16]. This interaction stimulates the catalytic activity of TdCAT1 in calcium dependent manner. Thus, identified TdCAT proteins were analyzed using Calmodulin target database server to identify the presence of Calmodulin binding domains (CaMBDs) in their structures. Our analyses showed that all identified catalases harbors at least 3 CaMBDs (Table 4). Biological significance of those domains must be investigated in plants.

Subcellular location of proteins is an important biological characteristic of those cell components [34]. Knowledge of proteins subcellular localizations is important to understand the mechanisms underlying protein cellular activities. Subcellular localization of different catalase proteins is already investigated. In Arabidopsis, catalase proteins are located in the main site of H_2_O_2_ production: peroxisomes. In rice, CAT proteins could be located in peroxisomes and cytoplasm [12]. Moreover, TdCAT1 and TmCAT1, the first isolated CAT proteins from *T. turgidum* and *T. monococcum,* respectively were located into peroxisomes and the deletion of the C-terminal portion of the protein (harboring PTS1 domain) leads to the translocation of those proteins into cytosol [15]. In bread wheat, TaCAT2A/B were localized in the cytoplasm and the nucleus [29]. In this work, in silico analysis of the identified proteins showed that TdCAT proteins could be located into peroxisome as revealed by Pannzer and Cello2GO server whereas LocTree and Wolf PSORT suggest that those proteins could also be located into cytoplasm, chloroplast and mitochondrion (Table 5).

Different key cellular processes (such as cell cycle control, signaling cascades, transcription regulation, and chaperone activity) are linked with disordered regions. The intrinsic flexibility of such proteins is an advantage to interact with different patterns with low affinity and high specificity [35]. TdCAT proteins presented small, disordered regions which varies from 14.5% in TdCAT3 to 16.25% to TdCAT5 (Supplementary Figure S3B). The presence of those regions in the TdCAT proteins suggest that those regions are related with the functions of catalase in cells and the importance of their metabolic roles in cellular regulations [36]. In another hand, durum wheat catalase proteins have no transmembrane region in their structures (Supplementary Figure S2).

Gene expression is controlled by the complex interaction of different cis-acting elements and trans-acting factors that participate in different pathways [26]. Previous investigations have confirmed that catalase genes could be induced by different treatments, such as salt stress [14,16], cold [10,29,37], drought [7,14,16,29,37,38], ABA [7,14,16,28,38], SA [16], and light [39].

As far as we know, no research has been carried out to study the cis-acting elements of *TdCAT* gene promoters. In this work, our analysis revealed the presence of different stress-responsive elements, such as anaerobic induction elements, meristem development elements, auxin responsive elements, abscissic acid responsive elements (ABRE), and light responsive, and defense and stress response (Figure 7 and Supplementary Figure S5). Interestingly, MYB-binding site (MBS) could be found in the promoter region of different catalase genes suggesting that some *TdCATs* could be regulated by the MYB transcription factor. All those founding suggest that *TdCAT* genes can be involved in plant maturation/growth and cell differentiation by acting as ROS regulators.

In the current study, we studied the transcript levels of 2 TdCATs (*TdCAT2* and *TdCAT3*) genes under extreme temperatures (heat and cold), ABA, NaCl, and mannitol treatments and in three different tissues (Roots, leaves and Stems) (Figure 8, Figure 9, Figure 10 and Figure 11). Those genes belong to different subgroups and all presented a constitutive expression pattern under normal development conditions with a low expression in stems for *TdCAT2*. In rice, *OsCatA* and *OsCatC* genes were essentially expressed in leaves in contrast to *OsCatB* gene which was expressed essentially in roots [40]. In hot peppers, the *CaCat2* gene was almost equally expressed in all tissues, whereas *CaCat1* gene was strongly expressed in vascular tissues and *CaCat3* was constitutively expressed in young seedlings and vegetative organs but with a low expression level [41]. In addition, seven different catalase genes were isolated from *Nicotiana tabacum* L., genome which were classified into 3 different groups [27]. According to tissue-specific analyses, NtCAT1-4 was expressed strongly in the shoots, whereas NtCAT5 and NtCAT6 were expressed strongly in the roots. In another hand, the circadian rhythms influenced NtCAT7 expression. During drought stress, NtCATs expression changed the most. Moreover, during cold stress, NtCAT5, NtCAT6, and NtCAT7 expression was increased, whereas it was down-regulated under drought and salt stress [27]. The plant’s reaction to environmental stress and H_2_O_2_ homeostasis in leaves is both regulated by SPCAT1 in the sweet potato (*Ipomoea batatas*) [42]. It’s interesting to note that ectopic CAT expression can influence CAT activity, as well as plant resilience to adversity. For instance, rice’s tolerance to low temperatures can be increased by the production of a wheat catalase gene [43]. Moreover, CAT activity can be induced by the ectopic expression of maize *CAT2* (*ZmCAT_2_*) in tobacco, enhancing pathogen resistance [44]. *TdCAT2* gene was upregulated under heat (38 °C), NaCl and mannitol but insensitive to cold stress application. Noteworthy, *TdCAT3* gene was found to be downregulated under cold stress conditions and upregulated under heat (38 °C), NaCl and mannitol suggesting that those genes could play specific roles in plants to respond to salt, drought and heat stresses. Interestingly, expression of *TdCAT2* and *TdCAT3* genes was rapidly increased when plants were subjected to ABA treatment suggesting that those catalase genes are closely related to the ABA signaling pathway in wheat. It is important to note that Gene ontology (GO) enrichment analysis showed that *TdCAT* genes are largely related to ROS response, cellular organelles, antioxidant enzymes and stimulus and stress response. The same results were showed for *Brassica napus* CAT [45]. In fact, *BnCAT* genes are related to stimulus responses, ROS response, and cellular organelles. Moreover, among the 14 identified genes, 10 *BnCAT* genes presented elevated expression levels in various tissues (roots, stem, leaf, and silique). Besides, *BnCAT1*, *BnCAT2, BnCAT3*, *BnCAT11*, *BnCAT12* and *BnCAT13* genes were highly upregulated by salinity, cold, and two hormones (ABA, and gibberellic acid (GA)) treatment, but not by drought and methyl-jasmonate (MeJA). Nevertheless, the roles of durum wheat *TdCATs* genes require further investigation. Our results open new windows for future investigations and provided insights into the *CAT* family genes in durum wheat.

## 4. Materials and Methods

### 4.1. Plant Materials, RNA Isolation and Data Sources

Durum wheat plants (*Triticum turgidum* ssp. *durum* var. Waha) [14] were grown in a growth chamber at 23–25 °C with an illumination of 16/8 h light/dark. Almost 50 seeds were treated with 30 mL of a 0.5% sodium hypochlorite solution for 15 to 20 min prior to incubation; they were then washed four times with 50 mL of sterile water. Seed germination was carried out in Petri dishes (11 cm wide, 11 cm long, and 2.5 cm high) containing a sheet of Whatman filter paper and a piece of sponge (to maintain moisture). Then, seeds were put in a greenhouse. The seeds were then given various stress treatments 10 days following incubation. For Mannitol and NaCl treatments, 10 days-old seedlings were supplemented with 150 mM mannitol or 150 mM NaCl, heat treatment (38 °C) and 5 mM ABA. Cold treatments were performed by transferring 10 days-old seedlings to a pre-cooled medium in a growth chamber at 4 °C. Wheat leaves, stems and roots were then collected 24 h after each treatment except for heat treatment were collected after 1, 6, 12 and 24 h of stress. Harvested leaves were immediately frozen in liquid nitrogen and then stored at −80 °C for RNA isolation.

Total RNA of *Triticum turgidum* subsp. *durum* cv. Waha was isolated using a specific Plant Total RNA Extraction Kit (BioFlux, Beijing, China). Samples were then reverse transcribed according to the instructions of ReverTra Ace qPCR RT Master Mix with gDNA Remover (TOYOBO, Osaka, Japan). The TtActin gene was used as internal Control. Moreover, each *TdCAT* genes (*TdCAT2* and *TdCAT3*) was analyzed with three technical replicates. A standard two-step PCR amplification procedure was performed as follows: 95 °C for 10 s, 59 °C for 10 s, and 72 °C for 20 s, 40 cycles. The cycle threshold (CT) value of the real-time PCR was further analyzed using the 2^−ΔΔCT^ method. Time 0 h (for each treatment) was normalized to 1. The primers used in this study for RT-qPCR were designed using NCBI Primer-BLAST tool (https://www.ncbi.nlm.nih.gov/tools/primer-blast/ (accessed on 1 May 2023)). The results of RT-qPCR were compared by one-way analysis of variance (ANOVA) followed by post-hoc Tukey’s test at *p* < 0.05.

### 4.2. Identification and Characterization Analysis of TdCAT Genes

The specific conserved domains of catalase PF00199 and pfam06628 were used as query to run Blastp in the genome of durum wheat. To confirm their acquisition to the two characteristics catalase domains, the durum wheat CAT proteins, originally obtained (25 sequence), were scanned by interpro (https://www.ebi.ac.uk/interpro/ accessed on 10 April 2023) [46], CD-Search (https://www.ncbi.nlm.nih.gov/Structure/cdd/wrpsb.cgi/ accessed on 10 April 2023) [47,48,49]; and HMMER (https://www.ebi.ac.uk/Tools/hmmer/ accessed on 10 April 2023) [50]. After removing the redundant sequences using decrease redundancy program (https://www.expasy.org/resources/decrease-redundancy; accessed on 10 April 2023) six TdCAT proteins were obtained and selected for further studies. The molecular weight (MW) and theoretical isoelectric point (pI) were computed using the ExPASY Compute pI/Mw tool (https://web.expasy.org/compute_pi/; accessed on 11 April 2023). The subcellular localization of TdCAT proteins was predicted using the PSORT: protein subcellular localization prediction tool (https://www.genscript.com/psort.html/; accessed on 14 April 2023), CELLO2GO (http://cello.life.nctu.edu.tw/; accessed on 14 April 2023) and LocTree3 (https://rostlab.org/services/loctree3/; accessed on 14 April 2023).

### 4.3. Phylogenetic Analysis of TdCAT Genes

To further clarify the evolutionary relationship between TdCATs proteins and CATs identified from other species, CAT protein sequences of *Arabidopsis*, *Nicotiana plumbaginifolia*, and rice were used to construct the phylogenetic tree. Full-length protein alignments were performed using MUSCLE [51], and a phylogenetic tree was constructed using MEGA-11 software (https://www.megasoftware.net/; accessed on 15 April 2023) using the maximum likelihood method with 1000 bootstrap.

### 4.4. Chromosomal Localization

*TdCAT* gene positions on chromosomes were visualized by using MG2C (http://mg2c.iask.in/mg2c_v2.1/; accessed on 12 April 2023) [52,53].

### 4.5. Conserved Motifs, and Gene Structure of TdCAT Genes

The intron–exon gene organization of *TdCAT* genes were analyzed using Tbtools. Moreover, MEME software (https://meme-suite.org/meme/ (accessed on 17 April 2023)) was used to identify the conserved motifs in TdCAT proteins. The protter database (https://wlab.ethz.ch/protter/start/, accessed on 17 April 2023) was used to study the presence of transmembrane domains in *TdCAT* genes structures. Finally, the presence of conserved CaMBDs was identified using the Calmodulin target database (http://calcium.uhnres.utoronto.ca/ctdb/no_flash.htm, [54] accessed on 17 April 2023).

### 4.6. The 2D and 3D Structures of Durum Wheat Catalase

Secondary structures of proteins were predicted by SOPMA (https://npsa-prabi.ibcp.fr/cgibin/npsa_automat.pl?page=npsa_sopma.html; accessed on 18 April 2023), whereas 3D structures were predicted using Alphafold online server (https://alphafold.ebi.ac.uk/, accessed on 20 April 2023).

### 4.7. Cis-Acting Element Analysis

To further identify the putative cis-regulatory elements of the promoter regions of the *TdCAT* genes, 1.5 kb upstream sequences of the genes were obtained from NCBI database (http://www.ncbi.nlm.nih.gov/, accessed on 20 April 2023). The different putative cis-regulatory elements of these sequences were further analyzed using PlantCARE databases (http://bioinformatics.psb.ugent.be/webtools/plantcare/html/ (accessed on 19 April 2023)). The diagram was visualized using TBtools software [55].

### 4.8. RNA Extraction and Quantitative Real-Time Reverse Transcription PCR (QRT-PCR)

Roots and leaves of durum wheat were separately used to extract total RNA (0.5 g of each tissue), using the RNeasy Plant Mini Kit (QIAGEN, Hilden, Germany). Extracted RNA was then purified from genomic DNA in presence of RNase free DNase set; QIAGEN), qualified using agarose gel, quantified using a nanodrop, then used for first-strand cDNA synthesis (GoScript Reverse Transcription System; Promega, Madison, WI, USA) with an oligo-dT primer. PCR reactions were realized in a 10 μL final volume tube in the presence of 3 μL cDNA (obtained from 40 ng of DNase-treated RNA), 0.5 μL of each primer of the *TdCAT* genes at 10 μM, 5 μL 2 x SYBR Green I master mix, and 1 μL of RNase-free water (Sigma, Burlington, MA, USA). The reactions consisted of an initial denaturation at 95 °C (5 min) followed by 40 cycles composed of 10 s at 95 °C, 20 s at 60 °C, and 30 s at 72 °C, then a melting curve (5 s at 95 °C, 1 min at 65 °C, and 5 min with the temperature increasing from 65 to 97 °C). Three biological repetitions were performed for each stress condition, with three technical repetitions for each sample. Melting curve analysis at the end of cycling was used to verify whether there was single amplification. At the end of the reaction, the threshold cycle (CT) values of the triplicate PCRs were averaged and used for transcript quantification. The relative expression ratio of the *TdCAT* genes was calculated by using the comparative CT method with the actin gene designed from the *T. aestivum* genome (actin_Fw: 50-TCC CTC AGC ACA TTC CAG CAGAT-3 and actin_Rv: 50-AAC GAT TCC TGG ACC TGC CTC ATC-30) as an internal expression standard [56]. The relative expression level was calculated from triplicate measurements based on the 2^−ΔΔCT^, where ΔΔCT = (CT, Target gene − CT, Actin) stressed − (CT, Target gene − CT, Actin). Relative expression ratios from three independent experiments (three biological repetitions) are reported.

### 4.9. Statistical Analysis

Data are reported as mean ± S.E. The results were compared statistically using Student’s *t* test, and differences were considered significant at *p* < 0.05.

## 5. Conclusions

Catalase proteins are important barriers that decompose the toxic H_2_O_2_ into water and O_2_^−^ to avoid cell death under unfavorable conditions. Even if catalase genes play crucial roles in wheat defense against different abiotic stress environment, the identification, isolation and molecular characterization of *TdCAT* genes remains also unclear. Here, different in silico analyses tools were investigated to enhance our comprehensive understanding of the catalase family in *Triticum durum* plants. In this work, six *TdCAT* genes were identified and clustered into three phylogenetic groups. Those genes were located in 3 different chromosomes. Other bioinformatic analyses showed that TdCAT proteins present highly conserved structures. Moreover, analysis of *TdCAT* gene promoters showed a myriad of cis-elements in the upstream of *TdCAT* genes. Such elements were found and may act in the gene expression to growth/development, hormones, and stress responses in durum wheat.

## Figures and Tables

**Table 1:** Features of *CATs* genes identified in *Triticum durum*. ObCATC: *Oryza brachyantha* Catalase C; TaCAT-1: *Triticum aestivum* Catalase-1; TcCAT-1: *Triticum diccocoides* Catalase-1; TmCAT: *Triticum monococcum* Catalase: HvCAT-2: *Hordeum vulgare* Catalase-2; AetCAT-2: *Aegilops taushii* Catalase-2.

	Locus/Protein Id	Gene Identifier/ORF Names/Locus Tag	Chr	Strand	EMBL ID	Star/End	N° Exon	N° Intron	Orthologs
TdCAT1-4B	WDD45561.1	TRITD_4Bv1G185220	Chr4B	-	LT934118.1	620612936….	6	5	ObCATC (XP_006649332.1; 92,07%)
									TaCAT-1 (NP_001392633.1; 99.8%)
						620616162			TcCAT-1 (XP_037426584.1; 99.52%)
									TmCAT- (QBZ38484.1; 98.37%)
TdCAT2	VAI41949.1	TRITD_6Av1G007920	Chr6A	+	LT934121.1	19325072….	3	2	TcCAT2 (XP_037449913.1, 96.96%)
						19327064			HvCAT-2 (XP_044952263.1, 96.56%)
									TaCAT-2 (XP_044407354.1; 96.56%)
TdCAT3	VAI53367.1	TRITD_6Bv1G012280	Chr6B	-	LT934122	33213343…	4	3	TcCAT-2 (XP_037454937.1; 88.06%)
						33215090			AetCAT-2 (XP_020150180.1; 87.85%)
									TaCAT-2 (XP_044407354.1; 87.45%)
TdCAT4	VAI53359.1	TRITD_6Bv1G012280	Chr6B	-	LT934122	33213343…	4	3	TcCAT-2 (XP_037454937.1; 86.84%)
						33215090			AetCAT-2 (XP_020150180.1; 87.04%)
									TaCAT-2 (XP_044410694.1; 86.64%)
TdCAT5	VAI10245.1	TRITD_4Bv1G185220	Chr4B	-	LT934118.1	620612936….	7	6	TaCAT-1 (XP_044376890.1 86.60%)
						620616162			TcCAT-1 (XP_037426584.1; 87%)
									AetCAT-1 (XP_020164896.1; 86.40%)
TdCAT6	VAI53365.1	TRITD_6Bv1G012280	Chr6B	-	LT934122	33213343…	4	3	TaCAT-1 (XP_044410694.1; 95.75%)
						33215090			TcCAT-1 (XP_037454937.1; 95.95%)
									AetCAT-1 (XP_020150180.1; 95.75%)

**Table 2:** General characteristics of TdCAT proteins using Protparam online software.

Gene Name	Locus/Protein Id	Length	Molecular Weight (MW)	Isoelectric Point (pI)	N-Glycosylation Site
*TdCAT1*	WDD45561.1	492	56,807.99	6.52	Asn-247
*TdCAT2*	VAI41949.1	510	58,611.25	6.29	Asn-247
*TdCAT3*	VAI53367.1	440	50,447.09	6.39	Asn-247
*TdCAT4*	VAI53366.1	464	50,316.01	6.97	Asn-198
*TdCAT5*	VAI10245.1	446	51,475.98	6.47	Asn-201
*TdCAT6*	VAI53365.1	475	54,695.84	6.35	Asn 228

**Table 3:** Physico-chemical analysis of TdCAT proteins sequences the ProtParam tool (http://web.expasy.org/protparam/, accessed on 2 April 2023).

Gene Name	Aliphatic Index	Gravy	Total Number of Negatively Charged Residues (Asp + Glu):	Total Number of Positively Charged Residues (Arg + Lys):	% of Alpha Helix	% Beta Turns	% Extended Strands	% Random Coils
*TdCAT-1*	69.15	−0.595	63	58	28.46	5.49	16.87	49.19
*TdCAT-2*	69.35	−0.483	69	62	26.4	5.69	15.29	52.55
*TdCAT-3*	69.52	−0.454	61	56	29.55	6.14	16.59	47.73
*TdCAT-4*	68.15	−0.552	60	59	27.79	5.24	13.21	53.76
*TdCAT-5*	68.21	−0.606	57	52	30.72	5.38	14.13	49.78
*TdCAT-6*	66.86	−0.518	65	59	27.16	4.00	14.32	54.53

**Table 4:** Number of identified CaMBDs in TdCAT1 proteins.

Gene Name	Number of Putative CaMBDs	Typical CaMBD	Position	IQ Motif	Position
*TdCAT-1*	4	3	48–79; 207–229	1	296–316
			462–486		
*TdCAT-2*	4	3	58–79; 214–229	1	296–316
			341–315		
*TdCAT-3*	3	2	59–77; 210–227	1	296–315
*TdCAT-4*	3	2	59–77; 241–260	1	287–305
*TdCAT-5*	4	3	66–87; 161–183	1	250–269
			417–437		
*TdCAT-6*	3	2	188–210; 322–342	1	277–296

**Table 5:** Localization of TdCAT1 proteins as predicted by three different databases.

Gene Name	CELLO2GO Results	LocTRee	Wolf PSORT	Pannzer2
*TdCAT-1*	Peroxisomal	Peroxisomal	Peroxisomal	Peroxisomal
*TdCAT-2*	Peroxisomal	mitochondrion	Cytoplasmic	Peroxisomal
*TdCAT-3*	Peroxisomal	mitochondrion	Cytoplasmic	Peroxisomal
*TdCAT-4*	Peroxisomal	Mitochondrion	Cytoplamsic	Peroxisomal
*TdCAT-5*	Peroxisomal	Peroxisomal	Chloroplast	Peroxisomal
*TdCAT-6*	Peroxisomal	Mitochondrion	Cytoplasm	Peroxisomal

**Table 6:** Gene ontology of different *TdCAT* in durum wheat.

Gene Name	Biological Process	Molecular Function
*TdCAT-1*	hydrogen peroxide catabolic process response to oxidative stress cellular oxidant detoxification response to oxygen-containing compound response to abiotic stimulus response to hormones intracellular nitric oxide homeostasis protein nitrosylation hydrogen peroxide biosynthetic process response to cadmium	catalase activity heme binding metal ion binding 5S rRNA binding protein binding structural constituent of ribosome
*TdCAT-2*	hydrogen peroxide catabolic process response to reactive oxygen species response to abiotic stimulus response to hormones cellular oxidant detoxification circadian rhythm response to acid chemical response to salt response to inorganic substance	catalase activity heme binding metal ion binding protein binding
*TdCAT-3*	hydrogen peroxide catabolic process response to oxidative stress cellular oxidant detoxification response to abiotic stimulus response to hormones circadian rhythm response to acid chemical response to inorganic substance response to salt	catalase activity heme binding metal ion binding protein binding
*TdCAT-4*	hydrogen peroxide catabolic process response to oxidative stress cellular oxidant detoxification response to oxygen-containing compound response to hormone response to abiotic stimulus	catalase activity heme binding metal ion binding
*TdCAT-5*	hydrogen peroxide catabolic process response to oxidative stress cellular oxidant detoxification response to oxygen-containing compound response to hormone response to abiotic stimulus intracellular nitric oxide homeostasis protein nitrosylation hydrogen peroxide biosynthetic process response to cadmium ion	catalase activity heme binding metal ion binding protein binding
*TdCAT-6*	hydrogen peroxide catabolic process response to reactive oxygen species response to abiotic stimulus cellular oxidant detoxification response to hormone circadian rhythm response to acid chemical response to inorganic substance response to salt	catalase activity heme binding metal ion binding protein binding

## Data Availability

The data generated and analyzed during this study are included in this article.

## Supplementary Materials

The following supporting information can be downloaded at https://www.mdpi.com/article/10.3390/plants12142720/s1, Figure S1: Phylogenetic analyses of the catalase proteins in *Triticum durum*. The phylogenetic tree was generated by the maximum-likelihood using MEGA 11; Figure S2: Analysis of the presence of signal peptides and the N-glycosylation sites in the TdCATs proteins sequences. (A) TdCAT1; (B) TdCAT2; (C) TdCAT3; (D) TdCAT4; (E) TdCAT5; (F) TdCAT6; Figure S3: Analyses of TdCAT proteins. (A) Number of predicted phosphorylation sites (PPS) in TdCAT proteins by using NetPhos 3.1 server. (B) Predicted percent disordered regions (DRs) of *T. durum* CAT proteins using PONDR online server; Figure S4: Identification of putative Calmodulin binding domains using the Calmodulin target database; Figure S5: Number of cis elements motifs in TdCAT promotors; Figure S6: QRT-PCR expression analysis of *TdCAT2* and *TdCAT3* genes under cold stress treatment (4 °C). Relative expression levels of TdCATs in response to cold treatment for non-stressed (0 h), 1 h, 6 h, 12 h, and 24 h at the 10 days old in the roots (A,D), stems (B,E) and leaves (C,F) of *TdCAT2* and *TdCAT3* genes, respectively. Data were normalized with *actin* gene, and vertical bars indicate standard deviation error. Different letters indicate significant differences at *p* < 0.05 according to one-way ANOVA and post-hoc Tukey’s test. NS means non stressed plants.
