# Supplementary material for: Catalase Gene Family in Durum Wheat: Genome-Wide Analysis and Expression Profiling in Response to Multiple Abiotic Stress Conditions
Source: Plants (Basel). 2023 Jul 21;12(14):2720. doi: 10.3390/plants12142720 (PMC10384705; doi:10.3390/plants12142720)
Supplement: Supplementary file 1 [file plants-12-02720-s001.zip › plants-2507964-supplementary.pptx]

## Slide 1
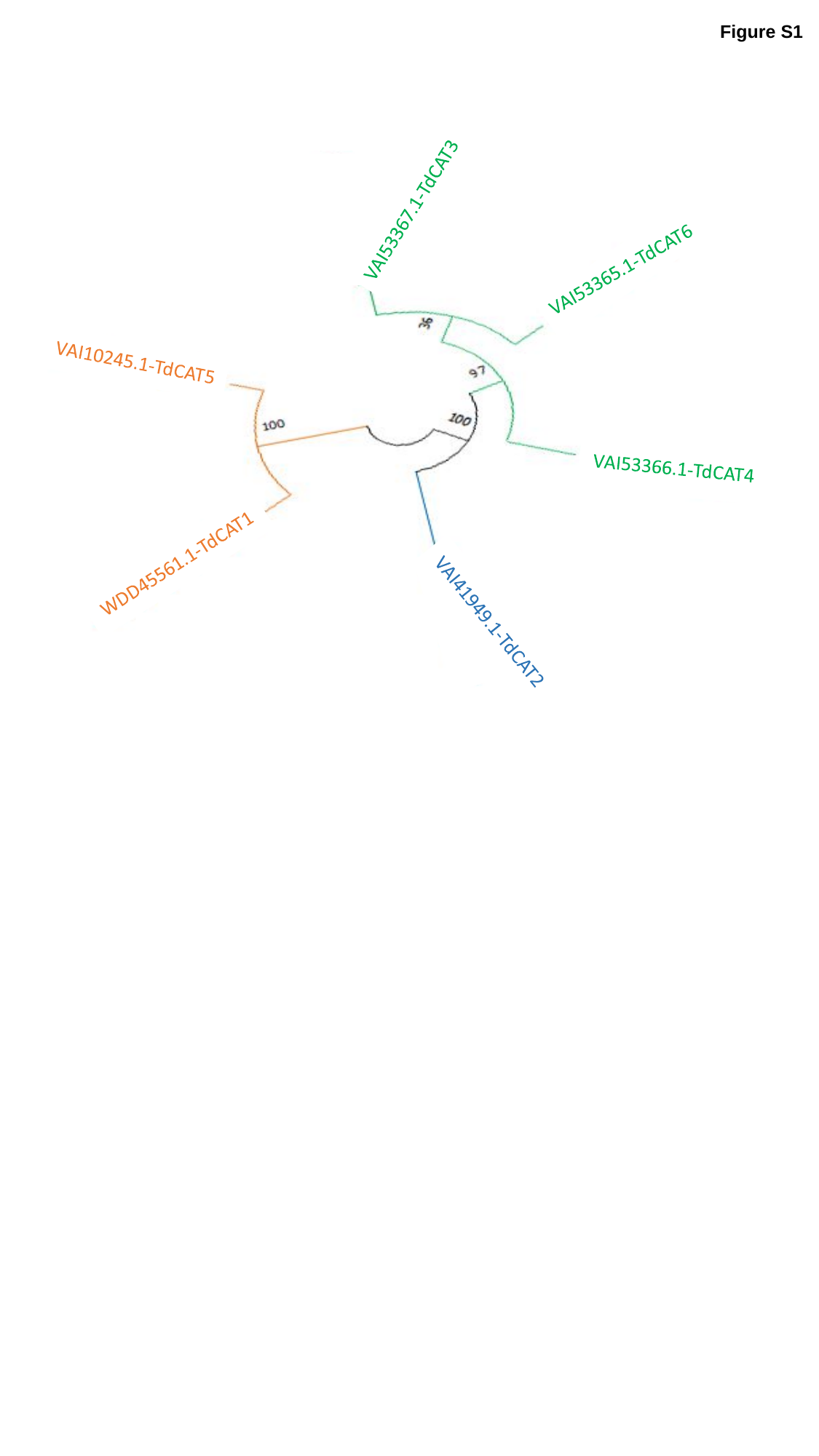

Figure S1

## Slide 2
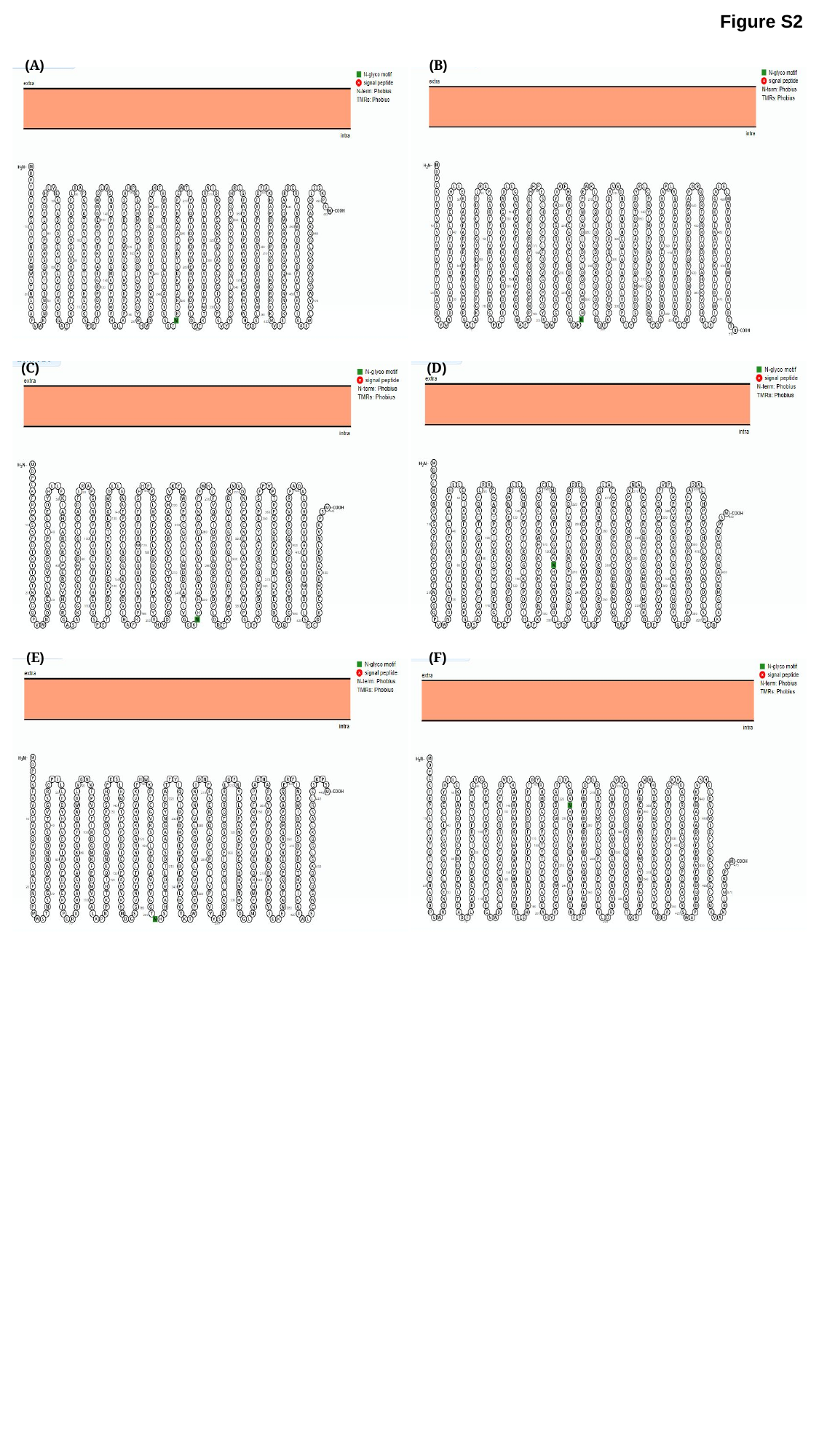

Figure S2
(A)
(B)
(C)
(D)
(E)
(F)

## Slide 3
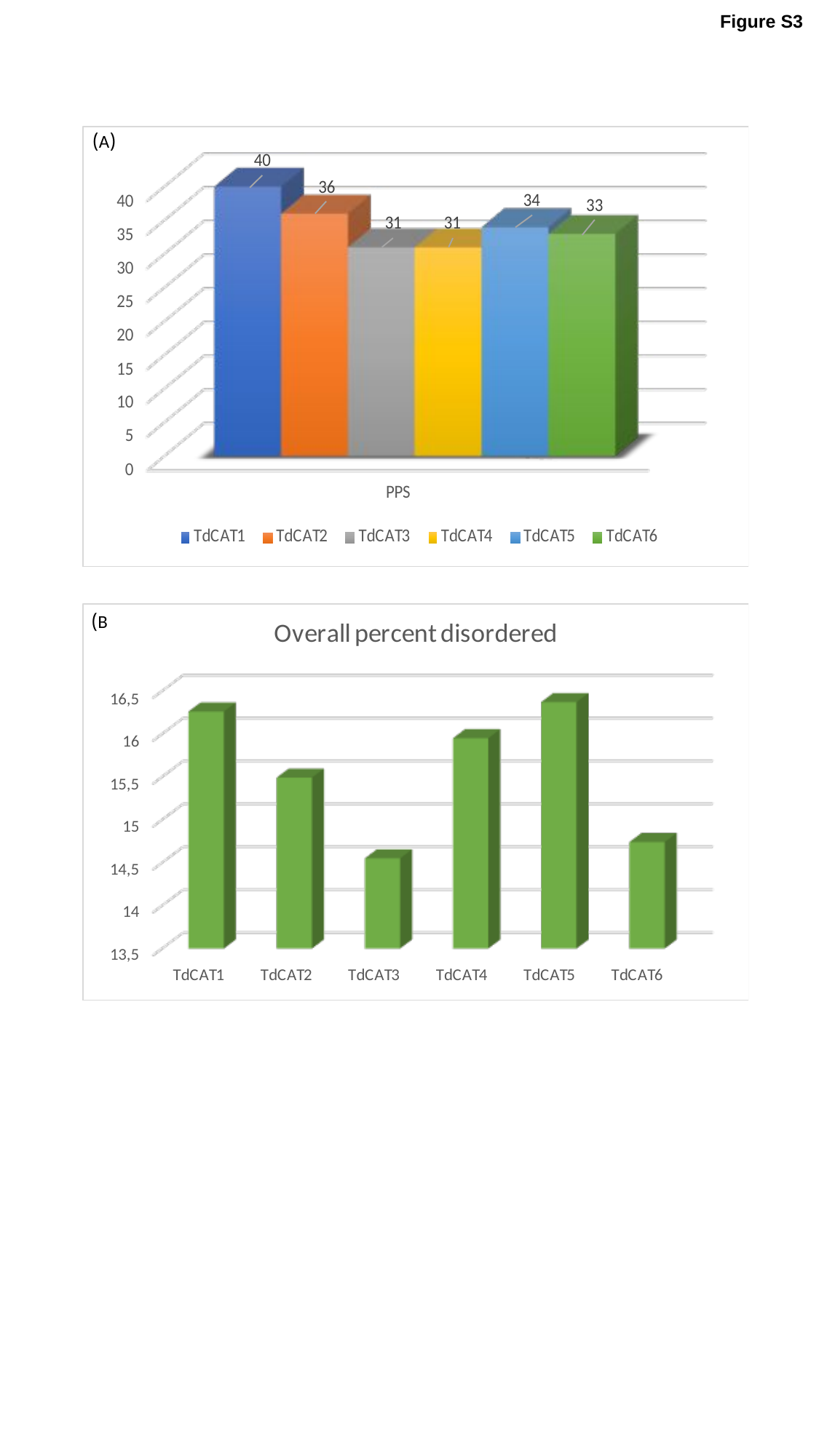

Figure S3
(A)
B)

## Slide 4
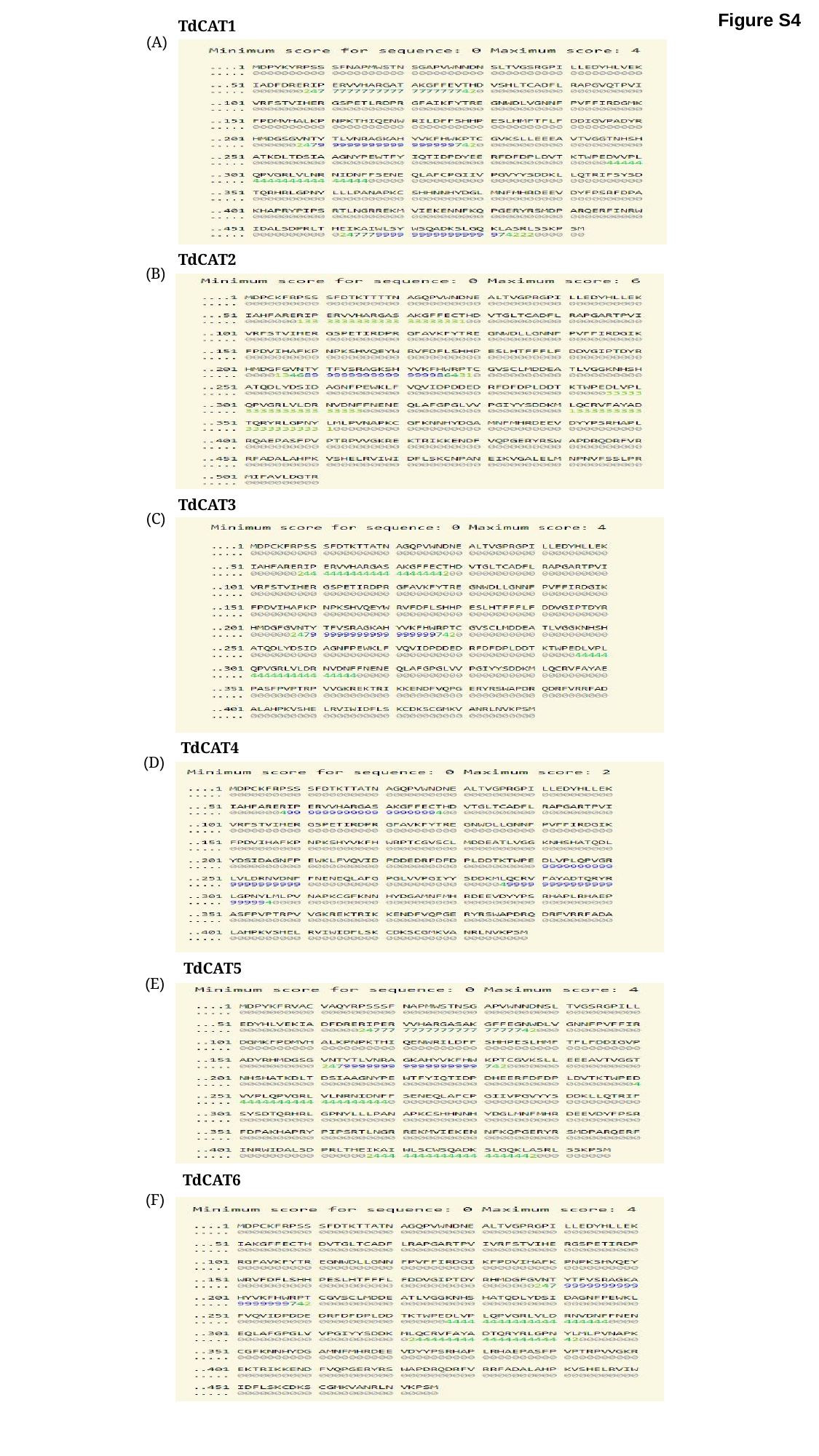

Figure S4
TdCAT1
(A)
TdCAT2
(B)
TdCAT3
(C)
TdCAT4
(D)
TdCAT5
(E)
TdCAT6
(F)

## Slide 5
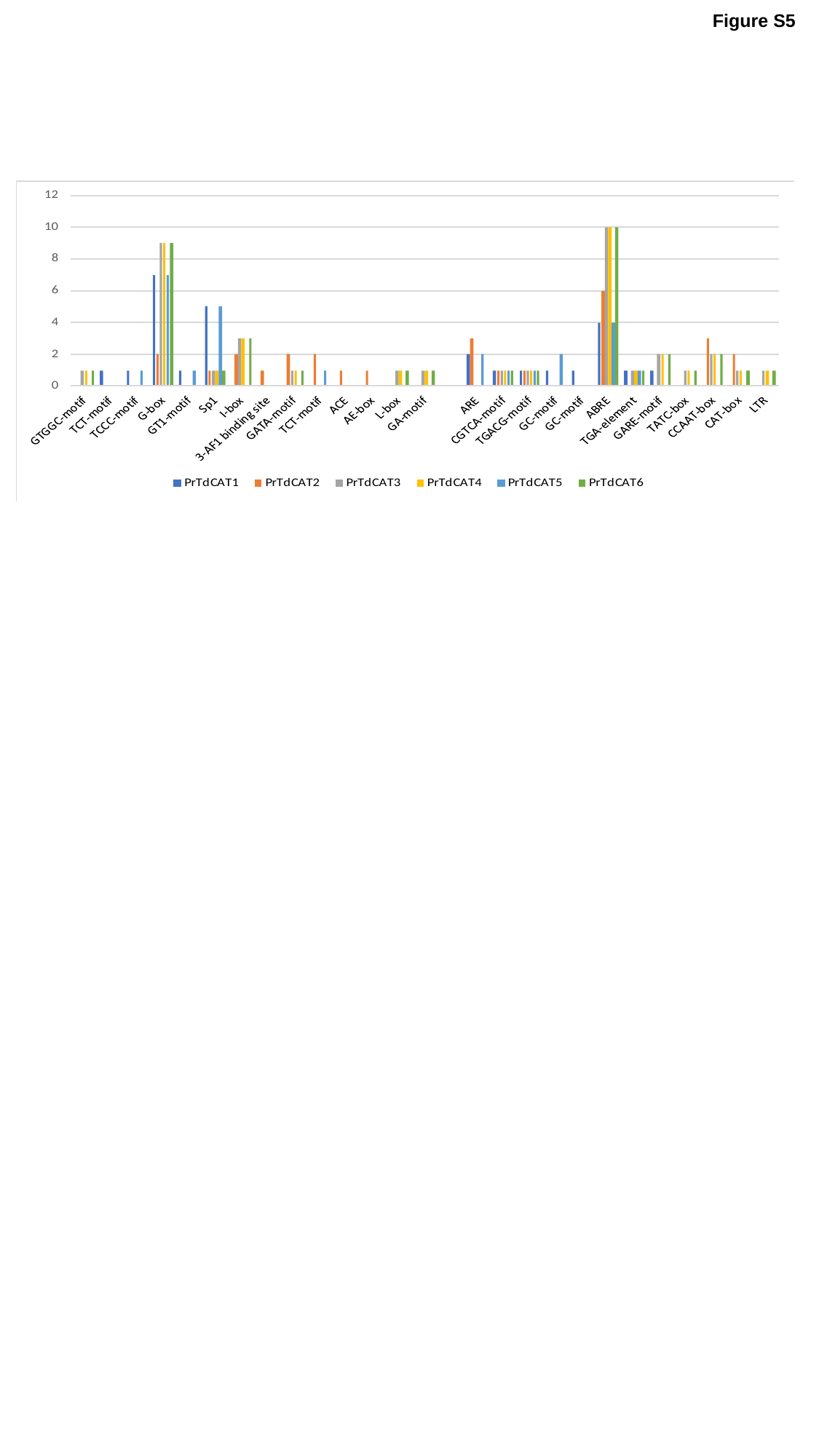

Figure S5

## Slide 6
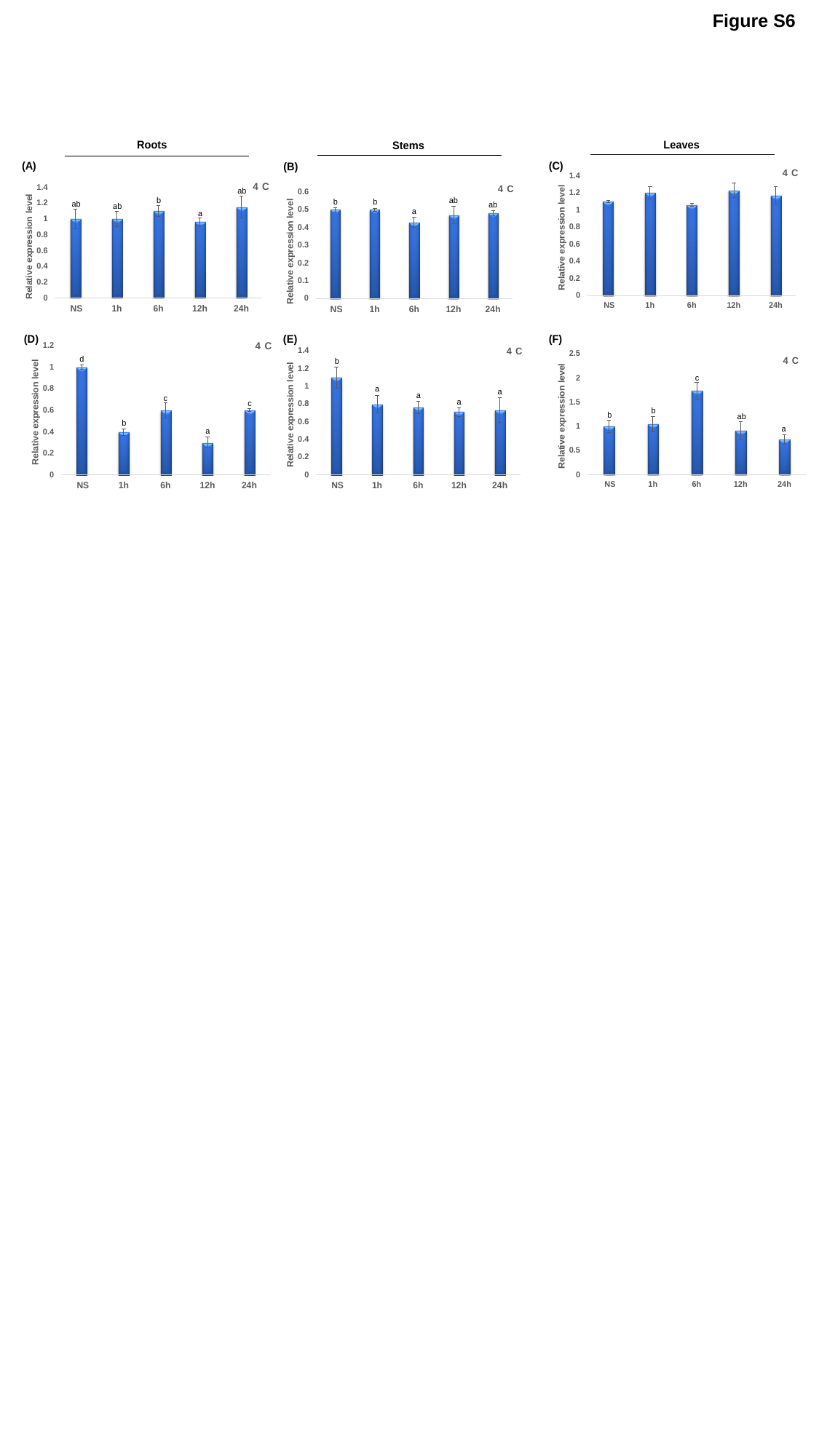

Figure S6
